# Codon optimality influences homeostatic gene expression in zebrafish

**DOI:** 10.1093/g3journal/jkae247

**Published:** 2024-10-24

**Authors:** Michelle L DeVore, Ariel A Bazzini

**Affiliations:** Stowers Institute for Medical Research, 1000 E 50th Street, Kansas City, MO 64110, USA; Stowers Institute for Medical Research, 1000 E 50th Street, Kansas City, MO 64110, USA; Department of Molecular and Integrative Physiology, University of Kansas Medical Center, 3901 Rainbow Blvd, Kansas City, KS 66160, USA

**Keywords:** codon optimality, zebrafish, mRNA, gene regulation, homeostasis, translation

## Abstract

The ribosome plays a crucial role in translating mRNA into protein; however, the genetic code extends beyond merely specifying amino acids. Upon translation, codons, the 3-nucleotide sequences interpreted by ribosomes, have regulatory properties affecting mRNA stability, a phenomenon known as codon optimality. Codon optimality has been previously observed in vertebrates during embryogenesis, where specific codons can influence the stability and degradation rates of mRNA transcripts. In our previous work, we demonstrated that codon optimality impacts mRNA stability in human cell lines. However, the extent to which codon content influences vertebrate gene expression in vivo remained unclear. In this study, we expand on our previous findings by demonstrating that codon optimality has a robust effect on homeostatic mRNA and protein levels in whole zebrafish during normal physiological conditions. Using reporters with nearly identical nucleotide sequences but different codon compositions, all expressed from the same genomic locus, we show that codon composition can significantly influence gene expression. This study provides new insights into the regulatory roles of codon usage in vertebrate gene expression and underscores the importance of considering codon optimality in genetic and translational research. These findings have broad implications for understanding the complexities of gene regulation and could inform the design of synthetic genes and therapeutic strategies targeting mRNA stability.

## Introduction

The regulation of gene expression is one of the most fundamental aspects of biological study. It requires stringent yet tunable control for development, metabolism, and aging, to react to changes in environment, to respond to infection and disease, and to maintain homeostasis (Signor and Nuzhdin 2018; Silva-Palacios *et al*. 2018; Carthew 2021). This regulation takes many forms, from epigenetic factors and transcriptional control to posttranscriptional mechanisms, including lncRNAs, tRNA abundance, microRNAs, and differential translation and degradation rates (Bazzini *et al*. 2012; Lykke-Andersen and Jensen 2015; Gulyaeva and Kushlinskiy 2016; Hanson and Coller 2018; Statello *et al*. 2021; Wu and Bazzini 2023). Generally, the amount of protein produced can be estimated by considering the level of transcription, the half-life of the mRNA, and the rate of translation (Presnyak *et al*. 2015; Medina-Muñoz *et al*. 2021; Diez *et al*. 2022).

**Fig. 1.**
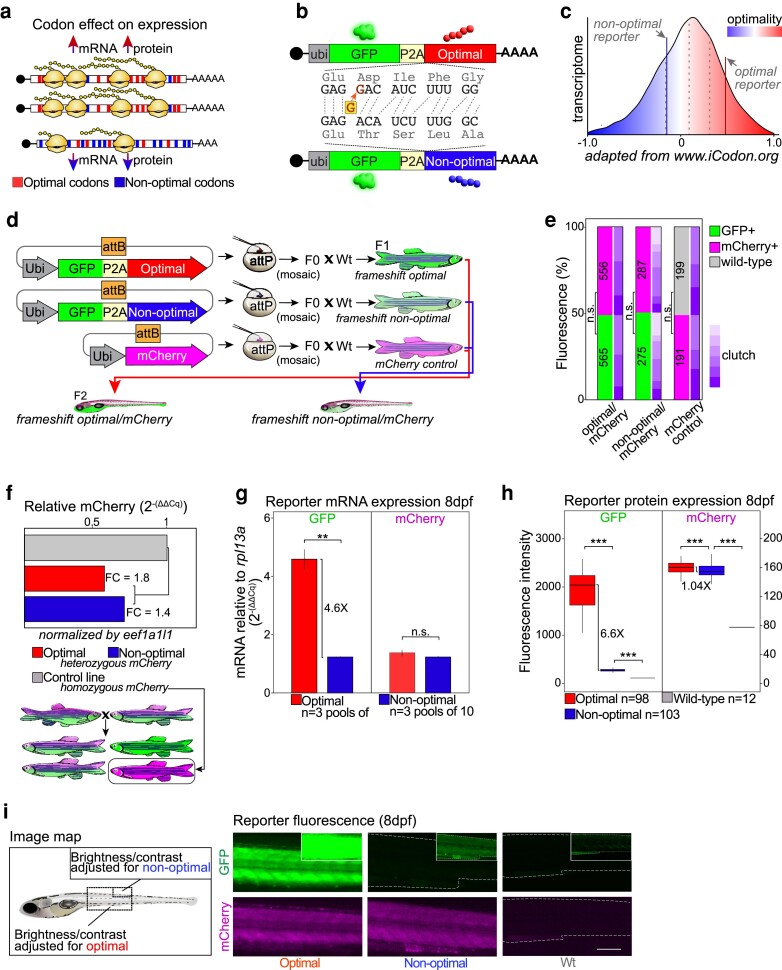
Codon optimality shapes homeostatic gene expression in zebrafish. a) Schematic illustration of the conceptual effect of codon optimality. An increased percentage of optimal codons are correlated with higher mRNA stability and increased translation efficiency. A greater portion of nonoptimal codons is associated with decreased mRNA stability and lower translation efficiency. b) Diagram depicting the reporter constructs inserted into the zebrafish genome. Each reporter is made up of the same nucleotide sequence apart from a single base insertion after the P2A in the optimal version. This mutation causes a frameshift that results in a change in optimality from being overall nonoptimal to being overall optimal, relative to the endogenous transcriptome. c) Histogram showing the relative optimality vs the number of transcripts for the zebrafish transcriptome. The optimality of the frameshift reporters is indicated, with the optimal scoring more optimal than 88% of endogenous gene and the nonoptimal just above the lowest quartile at 26%. The dotted gray line represents the mean optimality of the transcriptome (Diez *et al*. 2022). d) Schematic of the generation of transgenic zebrafish. Injection of *attB*-containing frameshift constructs with phiC31 integrase mRNA into embryos harboring a genomic *attP* landing site results in mosaic F0 progeny. Upon outcrossing to wild-type animals, F1 generation frameshift optimal, nonoptimal, and mCherry control are produced. Subsequently, the mCherry control line is crossed with each of the frameshift lines to create dual positive F2 offspring. e) Bar plot displaying the phenotypic frequency of GFP and mCherry expression in the frameshift optimal/mCherry, frameshift nonoptimal/mCherry, and mCherry control lines upon outcrossing to wild-type fish. The violet bars show the counts from individual clutches and the numbers describe total larvae counted. f) Schematic showing how homozygous mCherry animals were generated to use a copy number control. Relative mCherry expression of each line is shown in the bar plot. F2 generation frameshift optimal/mCherry fish were in-crossed, producing clutches containing 25% homozygous mCherry progeny. Genomic DNA from frameshift optimal, frameshift nonoptimal/mCherry, and homozygous mCherry fish was assayed via qPCR and normalized by housekeeping gene *eef1a1|1*. g) Bar plot showing the relative mRNA levels of GFP and mCherry in 8 dpf larvae. Frameshift optimal samples showed a GFP fold change of 4.6× compared with the frameshift nonoptimal samples, while there was no change in mCherry between frameshift optimal and frameshift nonoptimal fish. h) Box and whisker plot of fluorescence intensity measured in the trunk of 8 dpf larvae (the box indicates the IQR, the whiskers show the range of values that are within 1.5×IQR, and a horizontal line indicates the mean). i) Fluorescent images of 8 dpf larvae (right) and schematic indicating the region shown in the images and the visual adjustment used (left). The larger images are shown with brightness and contrast optimized for the frameshift optimal/mCherry line, while the smaller inset is the same image adjusted for the frameshift nonoptimal/mCherry. Dotted white lines outline the perimeter of images too dark to see. Scale bar = 150 mm. For all the pannels: n.s., not significant; *P < 0.05, **P < 0.01, ***P < 0.001 via Welch's 2-tailed *t*-test.

While many factors affect mRNA stability and translation efficiency, such as microRNAs, methylation, polyadenylation, and RNA-binding proteins (Bartel 2004; Bazzini *et al*. 2012; Van Nostrand *et al*. 2020; Jiang *et al*. 2021; Wu and Bazzini 2023), this work focuses on the regulatory role of the coding region (CDS) in influencing both mRNA and protein levels through a phenomenon known as codon optimality (Presnyak *et al*. 2015; Bazzini *et al*. 2016). Codon optimality describes the property of a codon to affect mRNA stability. It is important to note that codon optimality is different from the codon usage bias, which represents the frequency of a given codon. During translation, mRNAs enriched in certain codons confer increased stability and are thus called “optimal,” while the presence of others, termed “nonoptimal,” increase degradation (Fig. 1a). Additionally, it has been demonstrated through ribosome profiling embryos that mRNAs enriched in optimal codons exhibit higher translation efficiency, while conversely, those containing more nonoptimal codons show reduced efficiency (Bazzini *et al*. 2016; Fig. 1a). Therefore, the coding region not only dictates the amino acid sequence, it also communicates a regulatory code about mRNA half-life and translation efficiency—not through the nucleotide sequence, but rather by the specific codons that make-up the reading frame (Wu and Bazzini 2023). The codon optimality code, which evaluates the regulatory impact of all 61 coding codons, has been determined by examining the correlation between codon composition and mRNA stability profiles, whether for endogenous transcripts or for large-scale reporter libraries, with or without translation (Presnyak *et al*. 2015; Bazzini *et al*. 2016; Narula *et al*. 2019; Wu *et al*. 2019; Forrest *et al*. 2020). A common metric for ranking codons based on optimality is known as the codon stabilization coefficient, which is calculated as the Pearson correlation coefficient of the mRNA half-life and the frequency with which the codon occurs in the transcript (Presnyak *et al*. 2015). Using this scoring system, the overall optimality of a transcript can be determined based on the codon composition. However, more sophisticated predictors of mRNA stability based on the codon composition have been generated that can be useful for research and biomedical applications (Diez *et al*. 2022; Leppek *et al*. 2022).

Though the optimality of individual codons varies between species, codon-mediated regulation appears to be highly conserved (Wu and Bazzini 2023). In addition to earlier studies demonstrating codon optimality in human cell lines (Narula *et al*. 2019; Wu *et al*. 2019; Forrest *et al*. 2020) and vertebrate embryos (Bazzini *et al*. 2016; Mishima and Tomari 2016), it has been observed in *Escherichia coli* (Boël *et al*. 2016), yeast (Presnyak *et al*. 2015; Harigaya and Parker 2016), trypanosomes (de Freitas Nascimento *et al*. 2018), *Arabidopsis* (Chu and Wei 2021), *Caenorhabditis elegans* (Stenico *et al*. 1994), and *Drosophila* embryos (Bazzini *et al*. 2016) and cells (Zhao *et al*. 2017), and mosquito cells (Castellano *et al*. 2024). These studies provide valuable insights into how codon optimality can impact expression across various biological systems. However, the regulatory strength in vivo in vertebrate animals remains uncertain.

Previous research on codon optimality in vertebrates has focused on its effects during early development. While embryos serve as robust assays, caution is necessary when extending conclusions to postdevelopment stages. For example, soon after fertilization, embryos undergo a massive shift in expression dynamics as many maternally deposited transcripts are rapidly cleared, making way for zygotic expression during the maternal-to-zygotic transition (Tadros and Lipshitz 2009). The kinetics of this developmental program almost certainly never occurs again on such a scale during the lifespan of the organism. Additionally, because reporters were injected as mRNA, this method excludes any regulation that would have occurred prior to the synthesis of a mature mRNA molecule. Consequently, it is unknown whether the effect of codon optimality remains as influential in complex multicellular organisms beyond the unique environment in early embryogenesis.

In this study, we address these limitations by using transgenic zebrafish expressing reporters designed to provide a readout specific to codon optimality. By maintaining nucleotide sequence while altering codon content, we interrogate the role of codon optimality during vertebrate homeostasis. Our results show significant differences between optimal and nonoptimal reporters at both mRNA and protein levels.

## Materials and methods

### Zebrafish lines and husbandry

All experiments were performed in accordance with the Stowers Institute IACUC review board. Wild-type fish are from random parents of AB/Tubingen or Tüpfel long fin lines. Fish harboring the attP landing site are of the attP2B: Tg[phiC31.attP.2B, 20.8myl7: Enhanced Green Fluorescent Protein (EGFP)] line (Mosimann *et al*. 2013).

### mRNA in vitro transcription

Plasmids carrying phiC31 integrase (pcDNA3.1 phiC31, addgene #68310) were linearized with PvuII_HF (New England Biolabs) prior to in vitro transcription using the mMESSAGE mMACHINE Kit, following manufacturer's protocols. mRNA was quantified using Qubit Fluorometric Quantification.

### Image acquisition

Images were acquired on a Leica THUNDER Imager equipped with an M205 FA microscope stand, a Plan Apo 1.0× M Series objective and a DFC9000 GT sCMOS camera. A fluorescent signal from “GFP” labels was detected using an ET GFP filter set and “mCherry” labels were detected using an ET mCHER filter set. All image processing was done using ImageJ.

Zebrafish larvae were imaged submerged in embryo media in polystyrene petri dishes. Fluorescence measurements using the petri dish and media without fish were used to determine background fluorescence, which was subtracted from all measurements prior to further analysis. Each larva was measured separately by outlining a region of interest (ROI) across the trunk skeletal muscle compartment. Images of wild-type fish were collected and used to determine autofluorescence intensity.

Adult zebrafish were dissected to isolate the trunk skeletal muscles by removal of viscera and skin/scales. Samples were rinsed in 1% PBS to remove blood and adipose cells and placed on a glass microscope slide. Periodic application of 1% PBS was done as needed to prevent tissues from drying. Background fluorescence was removed from the images using the rolling ball algorithm in ImageJ, with a radius of 10,000 pixels. Each sample was quantified by averaging multiple square ROI across the area (*n* = 118 measurements across 3 optimal/mCherry fish, *n* = 95 measurements across 3 nonoptimal/mCherry fish, and *n* = 56 measurements across 2 wild-type fish).

### Vectors

All newly generated vectors were constructed using NEBuilder HiFi DNA Assembly Master Mix (NEB #E2621). PCR protocols are in Tables 1 and 2. After amplification, PCRs were digested with DpnI (NEB) for 15 min at 37°C in PCR buffer before extracting the amplicon from a 0.8% agarose/TAE gel using QIAquick Gel Extraction Kit. PCR protocols are listed in Tables 1 and 2.

**Table 1.** PCR protocol used to build the backbone of the constructs used in transgenesis.

Reagent	mL/reaction	Temp (°C)	Time (s)	Cycles
Nuclease free water	12	98	60	1
Phusion HF buffer (NEB)	4	98	10	
dNTPs (NEB)	0.5	56	30	20
Vector primer (IDT) F (10 mM): TGGTCCAGCCTGCTTTTTTG	1	72	240	
Vector primer (IDT) R (10 mM): GCACCCAGCTTTCTTGTAC	1	72	300	1
Phusion DNA polymerase (NEB)	0.5			
Template (pDestattB_ubi:EGFP pCM272, addgene #68339) (20 ng/mL)	1			
Total volume	20			

**Table 2.** PCR protocol used to build inserts of the constructs used in transgenesis.

Reagent	mL/reaction	Temp (°C)	Time (s)	Cycles
Nuclease free water	12	98	60	1
Phusion HF buffer (NEB)	4	98	10	
dNTPs (NEB)	0.5	56	30	20
Insert primer (IDT) F (10 mM): caaaaaagcaggctggacca-CAGGATCCTCTAACGGCG	1	72	240	
Insert primer (IDT) R (10 mM): tgtacaagaaagctgggtgc-GTGACTGGAGTTCAGACG	1	72	300	1
Phusion DNA polymerase (NEB)	0.5			
Template (in-house) (20 ng/mL)	1			
Total volume	20			

### Text editing

Some portions of the manuscript were edited for grammar, syntax, and punctuation using ChatGPT-3.5, an AI language model developed by OpenAI. The model was employed to ensure clarity and coherence in the text, adhering to the standards of scientific writing. The final version of the manuscript was reviewed and approved by all authors to ensure the accuracy and integrity of the content.

### Generation of transgenic zebrafish

Twelve random wild-type fish were crossed with 12 random *attP^2B^* line fish, and the resulting clutches were mixed together prior to injection. *attB_Ubi:GFP-optimal*, *attB_Ubi:GFPnonoptimal*, and *attB_Ubi:mCherry* plasmids were separately injected into 1-cell stage embryos at a concentration of 12 ng/mL with 100 ng/mL of capped phiC31 integrase mRNA in a total volume of 1 nL/embryo. Fifty F0 larvae from each line showing mosaic GFP expression, or mCherry expression in the case of the control line, and EGFP in the heart (*attP2B* line fish express EGFP driven by the cardiac cell-specific *myl7* promoter) were raised to maturity and outcrossed with wild-type animals. An F1 with ubiquitous reporter expression and an EGFP positive heart was raised and used to establish the frameshift optimal, frameshift nonoptimal, and mCherry control lines. Subsequent dual positive fish were made by crossing either frameshift line with the mCherry control lines. All transgenic strains are listed in Table 3.

**Table 3.** Transgenic zebrafish strains developed in this study.

Reference name	Full genomic nomenclature
Frameshift optimal	*Tg(attL, ubi:GFP-optimal/−, attR, −0.8myl7:EGFP)*
Frameshift nonoptimal	*Tg(attL, ubi:GFP-nonoptimal/−, attR, −0.8myl7:EGFP)*
mCherry control	*Tg(attL,ubi:mCherry/−, attR, −0.8myl7:EGFP)*
Frameshift optimal/mCherry	*Tg(attL, (ubi:GFP-optimal, attR/attL, ubi:mCherry, attR), −0.8myl7:EGFP)*
Frameshift nonoptimal/mCherry	*Tg(attL, (ubi:GFP-nonoptimal, attR/attL, ubi:mCherry, attR), −0.8myl7:EGFP)*
Homozygous mCherry	*Tg(attL, (ubi:mCherry/+, attR, −0.8myl7:EGFP)*

### Genomic DNA qPCR and RT-qPCR

All larvae were collected at 8 days postfertilization (dpf), euthanized using ice-cold MS-222, immediately transferred into homogenization buffer, ground using a motorized homogenizer, and then stored at −70°C. mRNA was extracted using the Promega Maxwell RSC simplyRNA Tissue Kit. The resulting isolated mRNA quality and concentration were evaluated on an RNA 6000 Nano chip using the Agilent 2100 Bioanalyzer system. Three hundred nanograms of RNA were used with random hexamers for each SuperScript IV Reverse Transcriptase cDNA synthesis reaction (Thermo Fisher Scientific). qPCR assays each used a 1:10 dilution of cDNA (primers listed in Table 4) and were pipetted using a Freedom EVO liquid handler (Tecan). Reactions were carried out in 384 well plates using a QuantStudio 7 Real-Time qPCR System. Primer sequences are summarized in Table 4.

**Table 4.** qPCR primer sequences.

Gene	Forward primer	Reverse primer
*eef1a1^a^*	GGGCAAGGGCTCCTTCAA	CGCTCGGCCTTCAGTTTG
*rpl13a^a^*	TCTGGAGGACTGTAAGAGGTATGC	AGACGCACAATCTTGAGAGCA
GFP	GGCCAGTTCTTCTTCCAGATAA	GTGTAATCCCTGCTGCTGTAA
mCherry	TGGGAAGCGTCATCAGAAAG	CATCGTAATGCCCTCCATCTT

^*a*^ *eef1a1* and *rpl13a* were used for normalization (Rassier *et al*. 2020).

## Results

To determine whether codon composition affects gene expression in vertebrates at homeostasis, we utilized a pair of reporters previously used in zebrafish embryos (Bazzini *et al*. 2016) and human cells (Wu *et al*. 2019) to provide a codon optimality-specific readout. Each reporter—1 optimal and 1 nonoptimal—encodes the same green fluorescent protein (GFP) coupled to the approximately equal length optimality segment by ribosome skipping sequence P2A (Fig. 1b and Supplementary Files 1–4). The P2A sequence allows the translation of 2 independent peptides from a single open reading frame (Kim *et al*. 2011). Therefore, GFP is not fused to the optimal or nonoptimal peptide, and consequently, the folding of the GFP should not be affected. Importantly, we introduced several synonymous mutations in the GFP sequence (used in both reporters) to neutralize the optimality compared with commonly used GFP variants. This adjustment is crucial because more frequently used GFP variants are highly enriched in optimal codons, which makes them inherently stable. The difference in codon optimality between the optimal and nonoptimal segments following the P2A may not have been sufficient to create a strong contrast in the overall codon optimality of the entire transcript if had used a highly optimal reporter. The second half of the reporter contains the optimality sequences, with both versions sharing the same nucleotide sequence, except for a single nucleotide insertion immediately after the P2A sequence in the optimal reporter. This insertion causes a frameshift that, while preserving the nucleotide sequence, changes the codon sequence. The optimal reporter is enriched with optimal codons, while the nonoptimal reporter is enriched with nonoptimal codons. This design ensures that the only difference between the 2 sequences is the codon composition, thereby isolating the effect of codon optimality. As a result, the constructs are referred to as “frameshift optimal” and “frameshift nonoptimal.”

The optimality of both reporters falls within the normal physiological range as calculated using iCodon (Diez *et al*. 2022), with the optimal and nonoptimal transcripts landing at an optimality >88 and 26% of endogenous zebrafish mRNA, respectively (Fig. 1c). This design allows for the exclusion of nucleotide sequence as a source of expression difference between the reporters. Specifically, given that the sequences in question are identical barring 1 nucleotide, the traditional regulatory mechanisms such as microRNAs and RNA-binding proteins, which typically interact with mRNA in a sequence-dependent manner, should equally affect both the optimal and nonoptimal reporters (Corley *et al*. 2020; Kilikevicius *et al*. 2022).

As a way of controlling for potential differences in transcription, we utilized phiC31 integrase, chosen for its high specificity and efficiency, and an existing transgenic strain already carrying a characterized *attP* landing site (Mosimann *et al*. 2013) to generate frameshift optimal and frameshift nonoptimal transgenic zebrafish lines, each with *ubiquitin* promoters (Mosimann *et al*. 2011; Fig. 1d). The ubiquitin promoters were selected for their ability to drive consistent expression across different tissues. This system targets vector insertion to a preexisting landing site, creating animals with transgenes at the same genomic locus (Mosimann *et al*. 2013). To further isolate the effect of codon optimality from transcription, we generated a third line harboring the fluorescent protein mCherry at the same locus. By crossing the mCherry control strain with the frameshift fish, the second color serves as an allelic internal control. This setup enhances the comparison between reporters by normalizing with mCherry expression (Fig. 1d). This strategy effectively addresses potential issues associated with using endogenous genes for normalization, which can exhibit significant variability in expression across different tissues.

To verify that a single copy was inserted, frameshift optimal/mCherry, frameshift nonoptimal/mCherry, and mCherry control fish were outcrossed to wild-type lines. A Mendelian 50:50 ratio of GFP:mCherry in the case of frameshift parents or mCherry:wt for the mCherry control line was verified through multiple clutches (frameshift optimal/mCherry GFP:mCherry *P* = 0.93, frameshift nonoptimal/mCherry GFP:mCherry *P* = 0.95, mCherry control mCherry:wt *P* = 0.86, Welch's 2-tailed *t*-test; Fig. 1e). Additionally, DNA quantification by qPCR showed that homozygous mCherry fish (obtained by in-crossing the control line and selecting mCherry positive/GFP negative animals) contained 1.8 times more mCherry DNA than frameshift optimal/mCherry and 1.4 times more than frameshift nonoptimal/mCherry animals (normalized by endogenous gene *eef1a1l1*), indicating that there is not likely >1 copy of the transgene present (Fig. 1f).

To determine whether the optimal reporter was differentially expressed compared to the nonoptimal reporter in the transgenic fish, mRNA was assessed via RT-qPCR. The optimal reporter accumulated 4.6 times more GFP mRNA compared with nonoptimal fish reporters at 8 dpf larvae (*P* = 0.01, Welch's 2-tailed *t*-test), while mCherry levels did not differ significantly between the lines (*P* = 0.32, Welch's 2-tailed *t*-test; Fig. 1g).

To quantify protein expression, we subjected 8 dpf larvae to fluorescence imaging and quantified the intensity in the trunk region (Fig. 1h). A 6.6-fold change in GFP intensity in the optimal line compared with the nonoptimal line (*P* > 0.001, Welch's 2-tailed *t*-test). There was a minor difference in the mCherry level between the lines, the frameshift optimal/mCherry being 1.04 times greater than frameshift nonoptimal/mCherry (*P* > 0.001, Welch's 2-tailed *t*-test).

Visually, the difference in reporter expression between the 2 lines was highly apparent. Adjusting the brightness and contrast settings for the frameshift nonoptimal line resulted in complete saturation when viewing the frameshift optimal animals. To facilitate better visualization, the appropriate settings for each are shown with the inset images adjusted for the nonoptimal samples and larger images adjusted for the optimal samples (Fig. 1i).

Moreover, similar results were observed in the skeletal muscle of adult fish as shown in Supplementary Fig. 1. Specifically, at the RNA level, the optimal reporter accumulated 11.4 times more GFP than the nonoptimal, relative to each respective mCherry (*P* = 2.12 × 10^−2^, Welch's 2-tailed *t*-test; Supplementary Fig. 1d). At the protein level, the mean GFP fluorescence in optimal adult muscle was 35 times greater than the mean GFP fluorescence in nonoptimal animals relative to each respective mCherry (*P* = 6.63 × 10^−3^, Welch's 2-tailed *t*-test; Supplementary Fig. 1e).

Together, these results demonstrate that codon optimality substantially influences homeostatic mRNA and protein levels in zebrafish.

## Discussion

Gene expression in multicellular organisms is influenced by numerous regulatory pathways, making it challenging to isolate the impact of a single pathway. In this study, we developed a model to specifically examine the influence of codon composition on both mRNA and protein levels. Our reporter pair, which shares nearly identical nucleotide sequences but differs in codon composition (optimal vs nonoptimal) due to a single nucleotide insertion causing a translation frameshift in an exogenous gene that has no biological function, allows us to measure differences in gene expression based solely on coding sequences. Although the protein products of the exogenous sequences were not analyzed in this study, similar types of reporters have been previously used to demonstrate the regulatory information encoded by codons through the injection of in vitro transcribed mRNA into zebrafish embryos, as well as by plasmid transfection into human and mosquito cells (Bazzini *et al*. 2016; Wu *et al*. 2019; Medina-Muñoz *et al*. 2021; Castellano *et al*. 2024).

Previous studies have measured the effects of codon optimality in vertebrates using cells, early embryogenesis, or transcriptomic profiles (Bazzini *et al*. 2016; Mishima and Tomari 2016; Narula *et al*. 2019; Wu *et al*. 2019; Forrest *et al*. 2020; Medina-Muñoz *et al*. 2021). However, the direct effect of codon optimality on the expression of individual genes at homeostasis in a multicellular organism remained unclear. Our findings reveal striking differences in GFP expression between transgenic fish expressing the reporter enriched in optimal codons and those enriched in nonoptimal codons. This suggests that codon optimality significantly affects gene expression at homeostasis in zebrafish.

The protein disparity between optimal and nonoptimal was even greater than the differences at the mRNA level. We have previously performed ribosome profiling in zebrafish embryos and found that genes enriched in nonoptimal codons are less efficiently translated compared with a control group of genes with similar RNA levels but not enriched in nonoptimal codons. Conversely, genes enriched in optimal codons exhibit higher translation efficiency compared with a control group with similar RNA levels but not enriched in optimal codons. These findings demonstrated that codon optimality can indeed influence translation efficiency in zebrafish (Bazzini *et al*. 2016). Furthermore, in both human cells and yeast, it has been recently reported that codon optimality can affect translation initiation and, consequently, overall translation efficiency (Barrington *et al*. 2023; Lyons *et al*. 2024). Moreover, and like our findings, in other systems (Presnyak *et al*. 2015), the differences at the protein or fluorescent level between genes enriched in optimal codons vs those (Gardin *et al*. 2014; Lareau *et al*. 2014; Hussmann *et al*. 2015; Bazzini *et al*. 2016; Weinberg *et al*. 2016; Wu *et al*. 2019).

Future research could build on this study by designing and testing additional constructs that feature enrichment in specific codons or incorporating different sets of synonymous mutations (Bazzini *et al*. 2016; Wu and Bazzini 2018). These reporters could help address how specific codon sequences and synonymous changes impact gene expression in differing biological contexts. Moreover, in the future, transgenic fish can be generated to get insight into the effects of codon composition between cell types, tissues, or across the stages of an organism's life. Additionally, the transgenic fish generated in this study could be beneficial in evaluating the effects of factors related to codon content by subjecting the fish to knockout and/or knockdown experiments (e.g. CRISPR-Cas9/13d; Moreno-Mateos *et al*. 2015; Kushawah *et al*. 2020), environmental or nutrition changes, or combining them with models of disease.

## Supplementary Material

jkae247_Supplementary_Data

## Data Availability

The original data underlying this manuscript can be accessed from the Stowers Original Data Repository at https://www.stowers.org/research/publications/libpb-2462. Supplemental material available at G3 online.
