# Supplementary material for: Codon optimality influences homeostatic gene expression in zebrafish
Source: G3 (Bethesda). 2024 Oct 24;14(12):jkae247. doi: 10.1093/g3journal/jkae247 (PMC11631405; doi:10.1093/g3journal/jkae247)
Supplement: jkae247_Supplementary_Data [file jkae247_supplementary_data.zip › Supplemental_Figure_1_Legend_G3-2024-405368.docx]

**Supplemental Figure 1**

1. Fluorescence microscopy images of adult frameshift reporter expression in skeletal muscle (TL = transmitted light). Dotted white lines outline the perimeter of images too dark to clearly see. Scale bars = 1mm.
2. Schematic indicating the region from which fluorescence intensity measurements were taken.
3. Graphic indicating the region and visual adjustment shown in the images.
4. Bar plot representing the mRNA quantification by RT-qPCR. The frameshift optimal line showed 11.4 times more GFP expression than the frameshift non-optimal (p-value = 2.12x10^-2^, Welch’s two-tailed t-Test) relative to endogenous gene *cdk2ap2,* while there was no significant difference between mCherry levels. Error bars = standard error of the means.
5. Box plot showing the fluorescent intensity of GFP and mCherry in the muscle of adult transgenic fish expressing frameshift optimal or non-optimal and mCherry reporters, as well as wildtype fish. Frameshift optimal fish showed a 35-fold increase in GFP expression over non-optimal (p-value = 6.63x10^-3^, Welch’s two-tailed t-Test, n=3 of each line), while they showed no significant difference in mCherry expression. Both transgenic lines had fluorescence significantly higher than the wild-type autofluorescence background.
