# Supplementary material for: Codon optimality influences homeostatic gene expression in zebrafish
Source: G3 (Bethesda). 2024 Oct 24;14(12):jkae247. doi: 10.1093/g3journal/jkae247 (PMC11631405; doi:10.1093/g3journal/jkae247)
Supplement: jkae247_Supplementary_Data [file jkae247_supplementary_data.zip › Supplemental_File_2_G3-2024-405368.docx]

#This is the plain text of the entire vector expressing the Non-optimal reporter.

GTTGTAAAACGACGGCCAGTGAATTGTAATACGACTCACTATAGGGCGAATTGGGTACGTACCGGGCCCCTAGTATGTATGTAAGTTAATAAAACCCATTTTTGCGGAAAGTAGATAAAAAAAACATTTTTTTTTTTTACTGCACTGGATCTAGCGGATCCGGGAATTGGGAATTCGTTAACAGATCTGCGGCCGCGGCTCGAGCAGGAAACAGCTATGACCATGATTACGCCAAGCTATCAACTTTGTATAGAAAAGTTGGCTCCGAATTCGCCCTTAAACTCGAGACCAGCAAAGTTCTAGAATTTGTCGAAACATTTATGTTATATATTTCCTGAAAAAAATTCTGAGTAAGTTCTTAAGTGTTATTGCCAGCAACATAAACAACAGACGGCAAAATGAATAAATGATAACAAAGCAGTAGGCTTAAATAAACCTAATTTTTATAGGCTGTTCTCTACAACCCTCAAACAGTGATTAGTTTTGTACTTATAAACTTGCCCTTTCATTCATATTTCAAGAAAATTGGTTCAGAAGATCTGGATATTCTAGCAGTTGTTCAAGCTCATGGAGGGATCAGTGACCTGATTCCACAATGACTAGGCCTAATCCAGAAATTAGATGACTGTCAACATAAAAAGGCACAGCACTCACTAGCTGCCCTATATATTTTATTATATTTTACATATATTATTTTATTTATTTAGCTCTGAGTGCTGTACTTTCTGGTTAAAGAAAACTGCTTACAACAGCTAACCTGTACTACCTCAGGCTCAGGGAATTTGGAACAGGTTTGTCTGGTTTGTTTCTTTAACCATGCATGCTTGTTTTCAACTATGGCAACACAGTCACATGGGACATTACAGAAATGATTTGTCGATGACATGCGACTTTTCTTTAATAAAGCGCAAAGATCCCAAAAAGCAAACTTTTAACAAAAATCATATAATTATATTTTCAATCCAGCTTTGTAGCAACTTTGTGCTGCTGTTCACTCAGCAACAGATAGTCAGTATAAGGTCAGTGTGTCTCAAAGCAGTGCCATCTGTTTCACACATTGCGTTCTATATATAAGTGTGCTGGTTGACACGACACTGTATAAGGCCTAGGCTAAAACACAAACAATGTAGAATGACACTGTGTTTTTTTTGTAAACAAATGTTGTTTTTGGTTAAACATCTTTGTGAAAACATCCTCCTGTCATGTATTTGCTATATTCAAATGTTAAACCCGTGCAGAATAGAACATATACAAAAAAAAACAACACAACACATTTTTAAACATTATTAAATATCAAGTATTGCTGGCAGTTCTGTTTCTGTTTTACAGTACCCTTTGCCACAGTTCTCCGCTTTTCCTGGTCCAGATTCCACAAGTCTGATTCACCAATAGCAAAGCGAATAAACAACCAAAGCAGCCAATCACTGCTTGTAGACTGTCCTGCGAGACCGGCCCATTCCAGCACATTCTGGAAACTTCCTTTATATGATAATTATAAATACATTTAAATTATTGATACAAAACATGTAATTCCTAGAACATAACCATAGCAATCATTAGTTTTCAGGGTAATTATGTATTTTTAGGATTTGACTGCGGAAAGATCTGGTCATGTGACGTCTCATGAACGTCACGGCCCTGGGTTTCTATAAATACAGTAGGACTCTCGACCATCGGCAGATTTTTCGAAGAAGAAGATCAGTTTCAGGAGCCGTACTGTTCCGTTTCAACGCAAATATTAACGGTAAGAGCGAATTTCCTAGTTTGTTTTCATGCCATTCTTTAAAACCATAGCGTATTACTTTAATTATAGTAAACTTTCGCTTTCTTTATTACAAGAGACGTTTTGTGTTGATTCTCCGCGGACATTTTCGGTCAGACAATCAGAAAATGACCGCGGAGGACCAGTAACTTGCATTACACGTAAGTTAAATCTTCGTGTATTAAAATGGTTAGGTTGTTAACGTCAAATAGGTTACCGTGTTTGCGTGTGATCAGGTTGGTTTTGTTAGATTTTTGTCAGTATTTTTAATTTATTTGTTTTAGTTTATTTATTTTTTTTGCTGAATCATAGTTTGTGAACAAAGAACCCGGATGTTACATACAGTACAGCCGCCATGTTACAGAGAGTTATAACTTAATCATTTTAAAAATAATTTTGCCTTACTTTTAGTTTGTCATGTTGAGAAATGAGGAAATGTTAAAATGAGGAAATATCCAATTAATTTAATATATCAAAATAATCCATGATTACAATGCACTGAACTGGAGAAAATTAAGATGTTTTCTAGTGTCATGAAACAAATGTAAGAGATGTACATTGTAGATGTTTTATGTCAAGAATTGGCTAGTTGATGCAGCATACTGGCGATACTCAGTTGTAATAACAGTAACGTTACATGTTAATAGACTACTGAGTATGCTGTTCTGTCTATGTATGCTCTGTAAGCTACGAGAAGGACTTTTTTAAACAGTAAAGGGTGCAATATTTTTACAAATTGAATTAAATAAAGGCTGTCTATTAAGTAATATGCTTGATATTTTTCTTACTTGATCGGAAATAAGAAAAAATATAAACGTTGTTGCTCTAAAAATCCTAGTTCAGTTTAGCCAACCACAAATACCTTTTTGTTCCTCCAACAGTTTTTTTTTCTTCTCTATAATATTTGGCAGTCTATAGTACTCCAAATGTTTCCCCACAGTCTAACTAATTGGTACAGCCAAAATCATGACACTTATTGCAATAATAATTTTGGTTCATTGGCATTGTTGATAGCCTGTGCCACTAATATGGTCGATTGATCATGCTTCAGGAAGAAAACTATATTGTTTGATGTAAGATTATTAAATCTTCACCTGCTTCCATTACAAACTATTCCCATCTTATTGAATTCTGGTATGTCTTAAAGGATTAGTTCACTTCCCAAATCAAAATTTACTTAGCCTTTTTTCATCCATGATCCCTTTTTTTCATCATTAATGAAGAAATTGTTTTTGAAAAAGTTTCAAGATTTTTTTCTCTATATTGTGGAGCTTGTTAGTTTAAAATTCCAAAATGCAATATGTGGCTTCAAATGGTTCTAAATGATCCCAGTCAAGGAATAACAGTCTTATCTAATGAAACCATTAGACCTTTAAAAATAAAAAAAATAAAAGTATTTATTTTTAAATGACTGAGTGATTAAGTTGAATTTCAGCGTTTCCTTACTGTGTAGAAGTCCTTCCTTACTGGCCCCACCCTTTGGTTCTCTGCCAATCTGCTACCTAATGTAATGTTGTGGAACATTATTATTCTTTATTTCTTAATTTTTTATTTTTTATTTTAAAACAATGTAAACTGCACAGATGTGCAGTTTGTTTAAAATGGCCAATGCTTTGGAAATGCATGACATAATTAGATTTCATGATGCACAAAGCCAAATCTCAGAGCTTGTGCAAAATGAGCTATCATTTCACTAGGTAAGACCCTAAATTTTCATATAGGATCATTTGGACAATTTTGCTGCAGGTAAAATGCATTCTATAGTCCACTGTCAGCCATTGTTTTGGATAGTATTTATTTTTCTCTACAAGTATAGTCAATAGTTTTCTATTATTTTAAAGGTTTGTAACATTTAAGGGTGACCAAATGCAAAGTAAAATTTCATTTTCGGGTGAACTATCTCGTTTAACATGGGAGAAGTGCAAAACATACATTATTGGCTAGAACATTGTAGTATTTTTTAAATGGAAATGTGTGATTGCTAATCTTACTTTGAATTTGTTTACAGGGATCCTTTAAGGGCGAATTCGACCCAAGTTTGTACAAAAAAGCAGGCTGGACCACAGGATCCTCTAACGGCGAAatgtcaaaaggagaagaacttttcacaggggttgttccaatacttgttgaacttgatggggatgttaatgggcataaattctcagtttcaggagaaggagaaggagaagcgacatatgggaaacttacacttaaattcatatgtacaacagggaaacttccagttccatggccaacacttgttacaacacttacatatggagttcaatgtttctcaagatatccagatcatatgaaacaacatgatttcttcaaatcagcaatgccagaaggatatgttcaagaaagaacaatattcttcaaagatgatgggaattataaaacaagagctgaagttaaatttgaaggggatacacttgttaatagaattgaacttaaaggaattgaatttaaagaagatgggaatatacttgggcataaacttgaatataattataattcacataatgtttatattatggcagataaacaaaaaaatgggattaaagttaattttaaaattagacataatattgaagatgggtcagttcaacttgcagatcattatcaacaaaatacaccaattggggatgggccagttcttcttccagataatcattatctttcaacacaatcagcgctttcaaaagaaccaaatgaaaaaagagatcatatggttcttcttgaatttgttacagcagcagggattacacatgggatggatgaactttacaaaGGAAGCGGAGCTACTAACTTCAGCCTGCTGAAGCAGGCTGGAGACGTGGAGGAGAACCCTGGACCTCTCGAGACATCTTTGGCTTTGAGAACTTTGAGGTCAACCGCTTTGAGCAGTTCAACATTAACTATGCAAACGAGAAGCTTCAGGAGTATTTCAACAAGCACATTTTCTCACTGGAGCAGCTTGAGTTCAGGAAGGTGCAGCATGAGCTGGAGGAGGCTCAGGAGAGAGCTGACATCGCCGAGTCCCAGGTCAACAAGCTCAGAGCTAAAAGCCGTGAATTTGGAAAGGGTAAAGAGGCTGAGGAGGCTGACTCCTTCGACTATAAGAGCTTCTTCGCCAAGGTTGGGCTGTCCGCCAAGACTCCTGATGACATCAAGAAGGCTTTTGCTGTCATTGACCAGGACAAGAGCGGCTTCATTGAGGAGGATGTGGAGGACTCCCTCTGTGAGGCCAAAGAGCTGTTCATCAAGACAGTCAAGCACTTCGGTGAGGACGCTGATAAGATGCAGCCTGATGAGTTCTTTGGGATTTTCGACCAGTTCTTGCGTATCCCCAAGGAGCAGGGCTTCCTGTCGTTCTGGAGAGGAAACTTGGCCAACGTCATCAGATACTTCCCCACACAGGCCCTCAACTTTGCTTTCAAGGACAAGTACAAGAAGGTCTTCGACATCACAGACAAGCTGGAGAACGAGCTGGCCAATAAGGAGGCTTTCCTCAGACAGATGGAGGAGAAGAACAGGCAGTTGCAGGAGCGGCTTGAGTTGGCAGAGCAGAAGCTCCAGCAGGTCTAGATACCCATACGATGTACCAGATTACGCATGAGATCGGAAGAGCACACGTCTGAACTCCAGTCACGCACCCAGCTTTCTTGTACAAAGTGGGGGATCCAGACATGATAAGATACATTGATGAGTTTGGACAAACCACAACTAGAATGCAGTGAAAAAAATGCTTTATTTGTGAAATTTGTGATGCTATTGCTTTATTTGTAACCATTATAAGCTGCAATAAACAAGTTAACAACAACAATTGCATTCATTTTATGTTTCAGGTTCAGGGGGAGGTGTGGGAGGTTTTTTCCAACTTTATTATACATAGTTGATAATTCACTGGCCGTCGTTTTACGGTACCTCTAGAGATCCACTAGTGTCGACGATGTAGGTCACGGTCTCGAAGCCGCGGTGCGGGTGCCAGGGCGTGCCCTTGGGCTCCCCGGGCGCGTACTCCACCTCACCCATCTGGTCCATCATGATGAACGGGTCGAGGTGGCGGTAGTTGATCCCGGCGAACGCGCGGCGCACCGGGAAGCCCTCGCCCTCGAAACCGCTGGGCGCGGTGGTCACGGTGAGCACGGGACGTGCGACGGCGTCGGCGGGTGCGGATACGCGGGGCAGCGTCAGCGGGTTCTCGACGGTCACGGCGGGCATGTCGACACTAGTTCTAGCCAGCTTTTGTTCCCTTTAGTGAGGGTTAATTTCGAGCTTGGCGTAATCATGGTCATAGCTGTTTCCTGTGTGAAATTGTTATCCGCTCACAATTCCACACAACATACGAGCCGGAAGCATAAAGTGTAAAGCCTGGGGTGCCTAATGAGTGAGCTAACTCACATTAATTGCGTTGCGCTCACTGCCCGCTTTCCAGTCGGGAAACCTGTCGTGCCAGCTGCATTAATGAATCGGCCAACGCGCGGGGAGAGGCGGTTTGCGTATTGGGCGCTCTTCCGCTTCCTCGCTCACTGACTCGCTGCGCTCGGTCGTTCGGCTGCGGCGAGCGGTATCAGCTCACTCAAAGGCGGTAATACGGTTATCCACAGAATCAGGGGATAACGCAGGAAAGAACATGTGAGCAAAAGGCCAGCAAAAGGCCAGGAACCGTAAAAAGGCCGCGTTGCTGGCGTTTTTCCATAGGCTCCGCCCCCCTGACGAGCATCACAAAAATCGACGCTCAAGTCAGAGGTGGCGAAACCCGACAGGACTATAAAGATACCAGGCGTTTCCCCCTGGAAGCTCCCTCGTGCGCTCTCCTGTTCCGACCCTGCCGCTTACCGGATACCTGTCCGCCTTTCTCCCTTCGGGAAGCGTGGCGCTTTCTCATAGCTCACGCTGTAGGTATCTCAGTTCGGTGTAGGTCGTTCGCTCCAAGCTGGGCTGTGTGCACGAACCCCCCGTTCAGCCCGACCGCTGCGCCTTATCCGGTAACTATCGTCTTGAGTCCAACCCGGTAAGACACGACTTATCGCCACTGGCAGCAGCCACTGGTAACAGGATTAGCAGAGCGAGGTATGTAGGCGGTGCTACAGAGTTCTTGAAGTGGTGGCCTAACTACGGCTACACTAGAAGAACAGTATTTGGTATCTGCGCTCTGCTGAAGCCAGTTACCTTCGGAAAAAGAGTTGGTAGCTCTTGATCCGGCAAACAAACCACCGCTGGTAGCGGTGGTTTTTTTGTTTGCAAGCAGCAGATTACGCGCAGAAAAAAAGGATCTCAAGAAGATCCTTTGATCTTTTCTACGGGGTCTGACGCTCAGTGGAACGAAAACTCACGTTAAGGGATTTTGGTCATGAGATTATCAAAAAGGATCTTCACCTAGATCCTTTTAAATTAAAAATGAAGTTTTAAATCAATCTAAAGTATATATGAGTAAACTTGGTCTGACAGTTACCAATGCTTAATCAGTGAGGCACCTATCTCAGCGATCTGTCTATTTCGTTCATCCATAGTTGCCTGACTCCCCGTCGTGTAGATAACTACGATACGGGAGGGCTTACCATCTGGCCCCAGTGCTGCAATGATACCGCGAGACCCACGCTCACCGGCTCCAGATTTATCAGCAATAAACCAGCCAGCCGGAAGGGCCGAGCGCAGAAGTGGTCCTGCAACTTTATCCGCCTCCATCCAGTCTATTAATTGTTGCCGGGAAGCTAGAGTAAGTAGTTCGCCAGTTAATAGTTTGCGCAACGTTGTTGCCATTGCTACAGGCATCGTGGTGTCACGCTCGTCGTTTGGTATGGCTTCATTCAGCTCCGGTTCCCAACGATCAAGGCGAGTTACATGATCCCCCATGTTGTGCAAAAAAGCGGTTAGCTCCTTCGGTCCTCCGATCGTTGTCAGAAGTAAGTTGGCCGCAGTGTTATCACTCATGGTTATGGCAGCACTGCATAATTCTCTTACTGTCATGCCATCCGTAAGATGCTTTTCTGTGACTGGTGAGTACTCAACCAAGTCATTCTGAGAATAGTGTATGCGGCGACCGAGTTGCTCTTGCCCGGCGTCAATACGGGATAATACCGCGCCACATAGCAGAACTTTAAAAGTGCTCATCATTGGAAAACGTTCTTCGGGGCGAAAACTCTCAAGGATCTTACCGCTGTTGAGATCCAGTTCGATGTAACCCACTCGTGCACCCAACTGATCTTCAGCATCTTTTACTTTCACCAGCGTTTCTGGGTGAGCAAAAACAGGAAGGCAAAATGCCGCAAAAAAGGGAATAAGGGCGACACGGAAATGTTGAATACTCATACTCTTCCTTTTTCAATATTATTGAAGCATTTATCAGGGTTATTGTCTCATGAGCGGATACATATTTGAATGTATTTAGAAAAATAAACAAATAGGGGTTCCGCGCACATTTCCCCGAAAAGTGCCACCTAAATTGTAAGCGTTAATATTTTGTTAAAATTCGCGTTAAATTTTTGTTAAATCAGCTCATTTTTTAACCAATAGGCCGAAATCGGCAAAATCCCTTATAAATCAAAAGAATAGACCGAGATAGGGTTGAGTGTTGTTCCAGTTTGGAACAAGAGTCCACTATTAAAGAACGTGGACTCCAACGTCAAAGGGCGAAAAACCGTCTATCAGGGCGATGGCCCACTACGTGAACCATCACCCTAATCAAGTTTTTTGGGGTCGAGGTGCCGTAAAGCACTAAATCGGAACCCTAAAGGGAGCCCCCGATTTAGAGCTTGACGGGGAAAGCCGGCGAACGTGGCGAGAAAGGAAGGGAAGAAAGCGAAAGGAGCGGGCGCTAGGGCGCTGGCAAGTGTAGCGGTCACGCTGCGCGTAACCACCACACCCGCCGCGCTTAATGCGCCGCTACAGGGCGCGTCCCATTCGCCATTCAGGCTGCGCAACTGTTGGGAAGGGCGATCGGTGCGGGCCTCTTCGCTATTACGCCAGCTGGCGAAAGGGGGATGTGCTGCAAGGCGATTAAGTTGGGTAACGCCAGGGTTTTCCCAGTCACGAC
